# Use and perceived effectiveness of non-pharmacological home remedies for digestive symptoms: a questionnaire-based survey among primary care patients

**DOI:** 10.1093/fampra/cmad046

**Published:** 2023-04-13

**Authors:** Paul Sebo, Yoann Gaboreau, Marie Morel, Dagmar M Haller, Hubert Maisonneuve

**Affiliations:** University Institute for Primary Care (IuMFE), University of Geneva, Geneva, Switzerland; Department of General Practice, University of Grenoble, Grenoble, France; TIMC UMR 5525, Grenoble Alpes University, Grenoble, France; University College of General Medicine, University Claude Bernard Lyon 1, Lyon, France; University Institute for Primary Care (IuMFE), University of Geneva, Geneva, Switzerland; University Institute for Primary Care (IuMFE), University of Geneva, Geneva, Switzerland; University College of General Medicine, University Claude Bernard Lyon 1, Lyon, France

**Keywords:** abdominal pain, constipation, diarrhoea, digestion, home remedy, nausea, primary care, stomach pain, vomiting

## Abstract

**Background:**

Patients frequently visit their primary care physician (PCP) for digestive symptoms. We aimed to compile a list of non-pharmacological home remedies (NPHRs) that patients frequently use and find effective so that PCPs can then propose them to their patients with various digestive symptoms.

**Methods:**

In this questionnaire-based survey on the use and perceived effectiveness of NPHRs for digestive symptoms, 50 randomly selected Swiss or French PCPs consecutively recruited 20–25 patients between March 2020 and July 2021. These patients were given a list of 53 NPHRs previously developed by our research team. They were asked whether they used them (Y/N) and whether they considered them to be ineffective, not very effective, moderately effective, or very effective in treating abdominal pain (14 NPHRs), bloating (2), constipation (5), diarrhoea (10), digestion trouble (12), nausea/vomiting (2) and stomach pain (8). We considered NPHRs to be perceived as effective if patients reported that they were moderately or very effective.

**Results:**

A total of 1,012 patients agreed to participate in the study (participation rate = 84.5%, median age = 52 years, women = 61%). The two most frequently used NPHRs were rice cooking water for diarrhoea (29% of patients) and prunes for constipation (22%). The perceived effectiveness of the NPHRs ranged from 82% (fennel infusions for abdominal pain) to 95% (bicarbonate for stomach pain).

**Conclusion:**

Our data could be useful to PCPs interested in proposing NPHRs to their patients suffering from digestive disorders, and more generally to all PCPs interested in learning more about patients’ use of NPHRs in primary care.

**Key messages**

°Half the patients used home remedies for digestive symptoms.

°The majority of users reported that home remedies were effective/very effective.

°The most frequently used home remedy was rice cooking water for diarrhoea (29%).

## Introduction

Patients frequently visit their primary care physician (PCP) for digestive symptoms, such as epigastric or abdominal pain, diarrhoea, constipation, or bloating.^1^ These may reflect organic conditions or functional disorders, particularly spastic colon and non-ulcer dyspepsia.^2^ To treat these symptoms, PCPs often resort to pharmacological drugs. This approach is not always cost effective and may be associated with multiple side effects.^3^

Non-pharmacological home remedies (NPHRs) can be proposed instead of or in addition to pharmacological drugs to treat a wide variety of symptoms. There are little published data on the use and/or benefit of NPHRs for digestive disorders. Three recent publications present evidence in favour of the effectiveness of the use of prunes or kiwi in constipation,^4,5^ and of kiwi in upper gastrointestinal disorders.^6^

Many patients would like their PCP to propose NPHRs, but PCPs rarely do so, mainly due to a lack of knowledge.^7–10^ Failure to meet patients’ expectations can alter the doctor–patient relationship and lead to poor adherence. Thus, it is important to increase PCPs’ knowledge of commonly used NPHRs for digestive symptoms, so that they can propose these alternatives more frequently.

In this primary care study in Switzerland and France, we aimed to compile a list of NPHRs that patients frequently use and find effective for digestive symptoms, so that PCPs can then open a discussion about them with their patients.

## Methods

This questionnaire-based survey took place between March/2020 and July/2021. Using official physician registers, we randomly selected PCPs practicing in Switzerland (Geneva area) or France (Lyon and Grenoble area) and invited them to participate by phone or email with the aim of recruiting 50 PCPs. If a PCP refused or if no response was received after three reminders, another PCP was randomly selected until the target sample size was reached. A total of 169 PCPs were contacted to reach the sample size (participation rate = 29.6%, Geneva: *n* = 15, Lyon: *n* = 18, Grenoble: *n* = 17).

Seven research assistants consecutively recruited 20–25 patients in the waiting rooms of the 50 PCPs who agreed to participate. To be included, patients had to visit their PCP for any kind of non-urgent health problem, be ≥18 years old, able to give informed consent and to read/understand all study materials in French. Reasons for exclusion were age<18 years, poor understanding of French and emergency consultations. Recruitment dates were agreed in advance between the research assistants and the PCPs, so that the recruitment process did not interfere with PCPs’ daily work.

To our knowledge, there is no validated instrument to explore patients’ use of NPHRs. We, therefore, developed an original questionnaire in several steps, which are described in detail in a previous study.^9^ The questionnaire consisted of two parts, the first focusing on socio-demographic items and self-estimated general health status,^11^ the second on NPHRs. It was to be completed by patients in the waiting room.

For the second part of the questionnaire, patients were given a list of 304 NPHRs to treat 79 common minor health problems (mainly symptoms). To develop this list, we recruited a random sample of 97 patients from 11 medical practices with whom we drew up a pre-list of NPHRs, which we then refined following discussions within our research team to arrive at the final list of 304 remedies used in the study. The supplementary material (https://osf.io/5JK49/) presents the questionnaire and the list of NPHRs for each medical condition (in French). In the present study, we limited the analysis to digestive symptoms only. Patients were asked whether or not they used NPHRs, and if they did, whether they considered them to be ineffective, not very effective, moderately effective, or very effective in treating abdominal pain (14 NPHRs), bloating (2), constipation (5), diarrhoea (10), digestion trouble (12), nausea or vomiting (2), and stomach pain (8). In the absence of a commonly accepted definition, we defined NPHRs as remedies that are not commercially available as drugs and do not require the external help of therapists.

Anticipating that the average prevalence of NPHR use would be about 60% with a 95% confidence interval of ±5% in absolute terms, and considering clustering (intra-class correlation coefficient = 0.05, 20 patients recruited on average per practice), the minimum sample size required was 720. To account for missing data we planned to recruit 1,000 patients.

Sociodemographic data were summarized as proportions (categorical variables) and medians and interquartile ranges (continuous variables). Data on NPHR use and perceived efficacy were summarized as proportions and 95% confidence intervals adjusted for intra-cluster correlations within medical practices. We considered NPHRs to be effective if patients reported that they were moderately or highly effective. We used the egen command in STATA to identify patients who used at least one of the NPHRs selected for the study. We then dichotomized patients into users/non-users and assessed whether the use of these remedies was associated with patient characteristics using univariable/multivariable logistic regressions, adjusting for intra-cluster correlations. The statistical significance was set at a two-sided *P* value of ≤0.05. All analyses were carried out with STATA 15.1 (College Station, TX).

## Results

A total of 1,012 patients out of 1,198 who were eligible agreed to participate (84.5%). Table 1 shows their main characteristics. Their median age was 52 years and there were 616 women (61.2%). Three hundred and sixty patients (35.6%) were recruited in Grenoble, 345 (34.1%) in Lyon, and 307 (30.3%) in Geneva. Only 100 patients (9.9%) were neither French (*n* = 690) nor Swiss (*n* = 219). The self-estimated general health status was very good for the majority of patients (excellent/very good = 31.7%, good = 52.0%, moderate/poor = 16.3%).

**Table 1.** Patients’ characteristics (*n* = 1,012 patients).

Characteristic	*n* (%)	Median [IQR]
Gender (*n* = 1,007)		
Female	616 (61.2)	
Male	391 (38.8)	
Age [years] (*n* = 1,006)		52 [31]
<40	295 (29.3)	
40–59	314 (31.2)	
≥60	397 (39.5)	
Region (*n* = 1,012)		
Grenoble (France)	360 (35.6)	
Lyon (France)	345 (34.1)	
Geneva (Switzerland)	307 (30.3)	
Location of the medical practice (*n* = 1,012)		
Urban zone	602 (59.5)	
Rural zone	410 (40.5)	
Nationality (*n* = 1,009)		
French	690 (68.4)	
Swiss	219 (21.7)	
Other	100 (9.9)	
Marital status (*n* = 1,004)		
Married or living as a couple	588 (58.6)	
Single	217 (21.6)	
Divorced or separated	127 (12.6)	
Widowed	72 (7.2)	
Work status (*n* = 1,009)		
Occupational activity	493 (48.9)	
Retired	324 (32.1)	
Student or apprenticeship/vocational training	61 (6.1)	
Recipient of unemployment (ALV) or invalidity (DI) benefits^a^	55 (5.5)	
Other	76 (7.4)	
Completed training (*n* = 1,009)		
University, FIT, UAS^b^	354 (35.1)	
Intermediate school^c^	479 (47.5)	
Compulsory schooling or no training/education	176 (17.4)	
Self-estimated general health status (*n* = 1,007)		
Excellent or very good	319 (31.7)	
Good	524 (52.0)	
Moderate or poor	164 (16.3)	

^a^ALV: unemployment insurance; DI: disability insurance.

^b^FIT: Federal Institute of Technology; UAS: University of Applied Sciences.

^c^Apprenticeship, vocational training, baccalaureate, or diploma from intermediate school.

On average, patients used one NPHR for digestive symptoms (median = 1; IQR = 5) and 509 patients (50.3%) used at least one of these remedies. Table 2 shows the 23 remedies used by at least 5% of patients and their estimated effectiveness. The two most frequently used NPHRs were rice cooking water for diarrhoea and prunes for constipation. Some remedies were used for several digestive symptoms, for example, Coke (5 symptoms) or fennel infusions (4 symptoms).

**Table 2.** Non-pharmacologic home remedies (NPHRs) used for digestive symptoms by at least 5% of primary care patients in the study, and their perceived effectiveness (*n* = 1,012 patients).

NPHRs shown by symptom and sorted by proportion of patients using them	Number of patients using the NPHR	Proportion of patients using the NPHR (95% CI)^a^	Number of patients who consider the NPHR effective or very effective among those who use it	Proportion of patients who consider the NPHR effective or very effective among those who use it (95% CI)^a^
Abdominal pain				
Hot water bottle on the abdomen	140	13.8 (11.8–16.2)	129	92.1 (86.2–95.7)
Self-massages of the abdomen	91	9.0 (7.2–11.3)	80	87.9 (79.4–93.2)
Coke	67	6.6 (5.0–8.8)	56	83.6 (73.9–90.2)
Fennel infusions	66	6.5 (4.3–9.7)	54	81.8 (72.1–88.7)
Hot baths	64	6.3 (5.0–8.0)	58	90.6 (80.7–95.7)
Bloating				
Fennel infusions	112	11.1 (8.7–14.0)	100	89.3 (82.5–93.7)
Constipation				
Prunes	227	22.4 (19.7–25.4)	203	89.4 (85.0–92.7)
Oat flakes and bran	51	5.0 (3.7–6.9)	45	88.2 (76.7–94.5)
Diarrhoea				
Rice cooking water	291	28.8 (25.4–32.4)	256	88.0 (83.5–91.4)
Coke	156	15.4 (12.9–18.4)	133	85.3 (80.0–89.3)
Bananas and shredded apples	94	9.3 (7.8–11.0)	89	94.7 (85.8–98.1)
Carrots	72	7.1 (5.6–9.1)	68	94.4 (85.8–98.0)
Digestion trouble				
Fennel infusions	124	12.3 (10.0–15.0)	107	86.3 (79.1–91.3)
Coke	119	11.8 (9.5–14.5)	106	89.1 (80.6–94.1)
Mint infusions	118	11.7 (9.4–14.4)	96	88.9 (80.6–93.9)
Chamomile infusions	108	10.7 (8.3–13.6)	96	88.9 (80.6–93.9)
Honey and lemon	71	7.0 (5.4–9.2)	61	85.9 (72.7–93.3)
Nausea or vomiting				
Coke	106	10.5 (8.5–12.8)	95	89.6 (80.3–94.8)
Stomach pain				
Hot water and lemon	95	9.4 (7.6–11.6)	86	90.5 (83.3–94.8)
Bicarbonate	83	8.2 (6.6–10.2)	79	95.2 (87.3–98.3)
Coke	70	6.9 (5.2–9.3)	60	85.7 (73.7–92.8)
Fennel infusions	62	6.1 (4.1–9.0)	51	82.3 (74.1–88.2)
Mint infusions	55	5.4 (3.9–7.6)	47	85.5 (72.6–92.9)

^a^The 95% confidence intervals (95% CI) were adjusted for intra-cluster correlations within medical practices.

The perceived effectiveness of the 23 NPHRs ranged from 81.8% (fennel infusions for abdominal pain) to 95.2% (bicarbonate for stomach pain). The lower limit of the 95% confidence interval was at least 72.1% (fennel infusions for abdominal pain).

Appendix #1 shows the associations between NPHR use for digestive symptoms and patient characteristics. In multivariable analysis, women and younger patients were more frequent users of NPHRs than men and older patients.

## Discussion

In summary, many patients attending medical practices in Switzerland and France use NPHRs to treat digestive symptoms and generally consider them to be effective. These results are in line with those of other studies in different contexts.^12,13^

The NPHR most used by the patients in the study was rice cooking water. To our knowledge, there are no published studies on this NPHR, perhaps because it is a typical remedy in our regions, but not on a global scale. More generally, few studies evaluated the efficacy of home remedies for digestive disorders,^4–6^ except perhaps where there is a potential for commercialization, as with fennel essential oils.^14^ It should be noted however that marketed remedies, such as essential oils, were not included in the definition of NPHRs used in our study. The medicinal use of fennel has been known for several centuries and recent studies have sought to evaluate its effectiveness and discover its active compounds.^15–18^

Considering the low efficacy of pharmacological drugs, such as phloroglucinol for abdominal pain,^19^ we believe that our data could be useful to PCPs interested in proposing NPHRs to their patients suffering from digestive disorders in addition to or instead of pharmacological drugs. All these remedies have the advantage of being perceived as effective by patients. They are inexpensive and likely cause few or no side effects (despite the absence of studies on this topic). In addition, the use of NPHRs can have a positive influence on the doctor–patient relationship, whilst reducing unnecessary costs. However, randomized controlled trials are needed in the future to scientifically prove their efficacy and safety. This would allow the widespread implementation of measures to promote the use of NPHRs in primary care, be it for digestive symptoms or more generally for a large number of minor health problems.

Our study has five main limitations. First, the results are not necessarily generalizable. The use of NPHRs seems to depend on language and cultural background, tradition, and the availability of natural resources.^20–22^ Second, as our study was observational, confounding factors could have biased our findings. In particular, the perceived effectiveness could have been overestimated. Third, the fact that this was a retrospective study and that the data were collected by questionnaire could also have led to information bias. However, the questionnaire was comprehensive and the list of NPHRs was previously compiled with the help of 97 patients. Fourth, there was a risk of selection bias among the PCPs participating in the study (participation rate = 30%). Finally, both patients and physicians were involved in creating the list of NPHRs on which this survey was based. Despite this, some of the (mostly newer) NPHRs, such as kiwi, were not cited in our list. This highlights the need for a dynamic approach in this field of research, considering the rapidly evolving use of NPHRs in various contexts.

## Supplementary Material

cmad046_suppl_Supplementary_Materials

## Data Availability

The data underlying this article will be shared on reasonable request to the corresponding author.
