# Supplementary material for: Use and perceived effectiveness of non-pharmacological home remedies for digestive symptoms: a questionnaire-based survey among primary care patients
Source: Fam Pract. 2023 Apr 13;41(3):373–7. doi: 10.1093/fampra/cmad046 (PMC11167983; doi:10.1093/fampra/cmad046)
Supplement: cmad046_suppl_Supplementary_Materials [file cmad046_suppl_supplementary_materials.zip › RGM_DIG_app1.docx]

Appendix #1. Associations between the use of non-pharmacological home remedies for digestive symptoms and patients’ characteristics (n=1,012 patients)

| Characteristic | Unadjusted OR (95%CI)^1^ | p-value | Adjusted OR (95%CI)^2^ | p-value |
| --- | --- | --- | --- | --- |
| Gender |  | <0.001 |  | <0.001 |
| Female | 1.8 (1.4-2.3) |  | 1.8 (1.4-2.4) |  |
| Male | 1 |  | 1 |  |
| Age [years] |  | <0.001 |  | <0.001 |
| < 40 | 2.6 (2.0-3.3) |  | 2.5 (1.9-3.3) |  |
| 40-59 | 2.4 (1.6-3.5) |  | 2.4 (1.6-3.5) |  |
| ≥ 60 | 1 |  | 1 |  |
| Location of the medical practice |  | 0.30 |  | 0.11 |
| Urban zone | 1 |  | 1 |  |
| Rural zone | 1.2 (0.9-1.6) |  | 1.3 (1.0-1.7) |  |
| Nationality |  | 0.31 |  | 0.67 |
| Swiss | 1 |  | 1 |  |
| Other | 1.2 (0.8-1.8) |  | 1.1 (0.7-1.6) |  |
| Completed training |  | 0.02 |  | 0.11 |
| University, FIT, UAS ^2^ | 1.4 (1.1-1.9) |  | 1.3 (0.9-1.8) |  |
| Other | 1 |  | 1 |  |
| Self-estimated general health status |  | 0.04 |  | 0.30 |
| Excellent or very good | 1.7 (1.1-2.7) |  | 1.1 (0.8-1.7) |  |
| Good | 1.5 (1.1-2.1) |  | 1.2 (0.9-1.7) |  |
| Moderate or poor | 1 |  | 1 |  |

^1^univariable logistic regression (adjusted for intra-cluster correlations within medical practices)

^2^multivariable logistic regression (adjusted for intra-cluster correlations within medical practices and for all variables listed in the table)

^3^FIT = Federal Institute of Technology; UAS = University of Applied Sciences
