# Supplementary material for: Use and perceived effectiveness of non-pharmacological home remedies for digestive symptoms: a questionnaire-based survey among primary care patients
Source: Fam Pract. 2023 Apr 13;41(3):373–7. doi: 10.1093/fampra/cmad046 (PMC11167983; doi:10.1093/fampra/cmad046)
Supplement: cmad046_suppl_Supplementary_Materials [file cmad046_suppl_supplementary_materials.zip › RGM_DIG_coverletter.docx]

| Dr. Paul Sebo  University Institute for primary care (IuMFE)  University of Geneva  Switzerland [paulsebo@hotmail.com](mailto:paulsebo@hotmail.com) | Editor-in-Chief  Family Practice  Letter transmitted online |
| --- | --- |

Geneva, 6^th^ February 2023

**Re: Use and perceived effectiveness of non-pharmacological home remedies for digestive symptoms: a cross-sectional study of primary care patients**

Dear Editor,

Many patients might be tempted to use non-pharmacological home remedies (NPHRs) instead of or in addition to pharmacological medications to relieve various ailments. However, primary care physicians (PCPs) rarely recommend NPHRs due to lack of knowledge in this field.

We conducted a questionnaire-based survey among primary care patients in Switzerland and France with the aim of compiling a list of NPHRs considered effective that PCPs could recommend to their patients suffering from various digestive symptoms. These symptoms were abdominal pain (14 NPHRs), bloating (2 NPHRs), constipation (5 NPHRs), diarrhea (10 NPHRs), digestion trouble (12 NPHRs), nausea/vomiting (2 NPHRs) and stomach pain (8 NPHRs).

Of the 1,198 eligible patients, 1,012 agreed to participate in our study (84.5%), 61% of whom were women. The two most frequently used NPHRs were rice cooking water for diarrhea (29% of patients) and prunes for constipation (22%). The perceived effectiveness of the NPHRs ranged from 82% for fennel infusions for abdominal pain to 95% for bicarbonate for stomach pain.

In summary, many patients recruited from medical practices in Switzerland and France used NPHRs to treat digestive symptoms and they generally considered them to be effective. Given the limited efficacy of pharmacological approaches and the potentially beneficial influence of the use of NPHRs on the patient-physician relationship, we believe that our data could be useful to PCPs interested in proposing NPHRs to their patients suffering from digestive disorders in addition to or instead of pharmacological drugs.

This manuscript has not been previously published and is not under consideration in any other media.

We thank you in anticipation for your attention to this paper.

Sincerely yours,

Dr. Paul Sebo
